# A Wideband Magneto-Electric (ME) Dipole Antenna Enabled by ME Resonance and Aperture-Coupled Excitation

**DOI:** 10.3390/mi16080853

**Published:** 2025-07-24

**Authors:** Hyojin Jang, Seyeon Park, Junghyeon Kim, Kyounghwan Kim, Sungjoon Lim

**Affiliations:** Department of Intelligent Semiconductor Engineering, Chung Ang University, Seoul 06974, Republic of Korea; abhyojincd0719@cau.ac.kr (H.J.); cosmosdus@cau.ac.kr (S.P.); wjdgus6748@cau.ac.kr (J.K.); kevin9711@cau.ac.kr (K.K.)

**Keywords:** wideband, aperture-coupled excitation, magneto-electric (ME) dipole antenna

## Abstract

In this study, we propose a novel wideband aperture-coupled magneto-electric (ME) dipole antenna that achieves enhanced bandwidth by simultaneously leveraging ME resonance and aperture-coupled excitation. Building upon the conventional ME dipole architecture, the antenna integrates a pair of horizontal metal patches forming the electric dipole and a pair of vertical metal patches forming the magnetic dipole. A key innovation is the aperture-coupled feeding mechanism, where electromagnetic energy is transferred from a tapered microstrip line to the dipole structure through a slot etched in the ground plane. This design not only excites the characteristic ME resonances effectively but also significantly improves impedance matching, delivering a markedly broader impedance bandwidth. To validate the proposed concept, a prototype antenna was fabricated and experimentally characterized. Measurements show an impedance bandwidth of 84.48% (3.61–8.89 GHz) for $S_{11}$ ≤ −10 dB and a maximum in-band gain of 7.88 dBi. The antenna also maintains a stable, unidirectional radiation pattern across the operating band, confirming its potential for wideband applications such as 5G wireless communications.

## 1. Introduction

As wireless communication technologies evolve, 5G and 6G systems are receiving increasing attention. Accordingly, antenna topologies ranging from simple configurations to complex, high-performance platforms are under active development. Compared with 5G, 6G will require a larger number of antennas, wider frequency bands, more flexible operation and improved performance. These demands call for low-power, high-performance chips, compact multiband antennas and advanced beam-forming techniques [1,2,3].

The conventional patch antenna—until recently the most widely adopted design—offers directional radiation, a simple structure and ease of fabrication, and therefore serves many applications [4]. Its restricted bandwidth and gain, however, have prompted the exploration of alternative structures, including array antennas and cavity-backed microstrip patch antennas [5,6]. To obtain a unidirectional radiation pattern together with wider bandwidth, Luk and Wong introduced in 2006 a new wideband complementary antenna known as the magneto-electric (ME) dipole antenna [7]. Formed by an L-probe, a vertical quarter-wave short-circuited patch and a planar dipole, the ME dipole exhibits low cross-polarization, low back radiation and symmetrical E- and H-plane radiation patterns. Extending its impedance bandwidth remains essential for stable operation over an enlarged frequency range.

There are various methods to increase the impedance bandwidth of an antenna. A fundamental strategy is to optimize the antenna’s geometry. By adjusting physical dimensions such as the aperture size or the length and width of the dipole structure, the impedance bandwidth can be controlled [8]. Broadband feeding techniques also represent a key method for improving impedance matching over wide operating bandwidths. At present, three major feeding methods are widely used for ME dipole antennas: probe, transmission line and aperture excitation [9]. In probe excitation, the position and structure of the probe can be modified to control the excitation waveform and optimize antenna performance [9,10,11,12]. However, this method typically results in more complex probe designs, which increases fabrication difficulty. Transmission line feeding facilitates the implementation of array antennas and makes it easier to achieve high gain [13]. Nevertheless, due to the complexity of large-scale designs, it becomes difficult to miniaturize the structure. Among these methods, aperture excitation not only effectively excites the resonance mode through a slot etched in the ground plane but also introduces an additional resonance point, enabling the utilization of multiple resonances. Furthermore, it offers easier fabrication and leads to simpler design layout [14,15,16]. By leveraging these various feeding techniques, engineers can achieve optimal performance across a wide frequency range.

Achieving wide impedance bandwidth is a critical objective in modern antenna design. However, beyond simply attaining a broad bandwidth, it is equally important to optimize impedance matching throughout the entire operating frequency range to ensure stable performance. To this end, techniques such as tapered feed lines, baluns and stubs are often employed to improve impedance matching. Additionally, recent studies have proposed enhancing the gain and radiation efficiency of ME dipole antennas through approaches such as metamaterial stacking, the incorporation of additional reflectors, or the use of low-temperature co-fired ceramic (LTCC) technology [9,17,18,19,20]. Although these methods can be effective, they often lead to bulky configurations and introduce parasitic elements that complicate the fabrication process.

The key contribution of this study lies in the proposal of a novel ME dipole antenna structure that achieves wideband performance while maintaining structural simplicity. Conventional probe-fed methods often involve complex designs and present challenges in controlling resonance modes. In contrast, this study adopts an aperture excitation technique using a tapered feed line, which naturally induces additional magnetic resonance within a simplified structure. This approach clearly distinguishes itself from previous work by effectively utilizing multiple resonances without the need for extra radiating elements. Notably, the proposed antenna achieves a wide impedance bandwidth of 84.48% without resorting to complex array configurations or multilayer metamaterials, while maintaining a stable unidirectional radiation pattern across the entire operating frequency band.

The remainder of this paper is organized as follows. Section 2 details the antenna geometry and design methodology, with a focus on the broadband impedance matching process. Subsequently, the magneto-electric resonant modes at each resonance frequency are analyzed from an electromagnetic field perspective. Section 3 presents the fabrication and measurement results of the optimized antenna prototype. Finally, Section 4 and Section 5 provide discussion and conclusions, respectively.

## 2. Materials and Methods

### 2.1. Antenna Geometry

**Figure 1:**
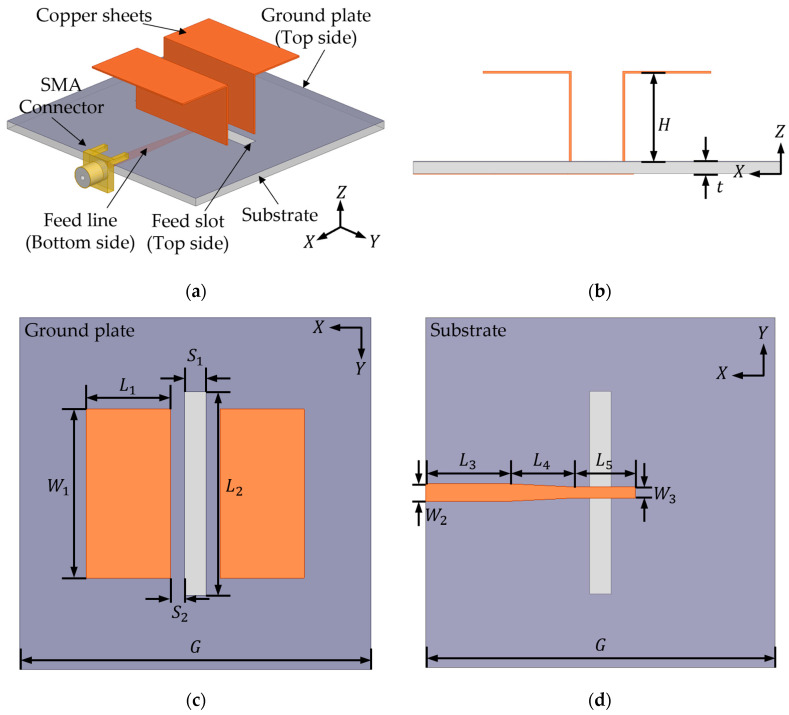
Geometry of the proposed ME dipole antenna: (**a**) three-dimensional view of the antenna; (**b**) side view of the antenna; (**c**) top view of the antenna; (**d**) bottom view of the antenna.

Figure 1 presents the geometry of the proposed ME dipole antenna from multiple perspectives. Electromagnetic energy is transferred from a tapered microstrip line located on the bottom of the substrate to a pair of copper sheets via a slot etched into the ground plane. The substrate material is FR4, with a thickness of 1.6 mm and a dielectric constant of 4.4. The overall dimensions of the proposed antenna, optimized through simulation, are summarized in Table 1. The total size of the antenna is 50 × 50 × 13.6 mm^3^.

The antenna was simulated and optimized using the full-wave electromagnetic solver Ansys High-Frequency Structure Simulator (HFSS). In the HFSS simulation, the substrate was modeled with a relative permittivity of 4.4 and a loss tangent of 0.02. The copper sheets were assigned a bulk conductivity of 5.8 × 10^7^ S/m. Additionally, a connector was included in the model, with a wave port assigned at its excitation interface. The simulation settings were as follows: the number of solved elements in the final adaptive pass was approximately 24,814, ensuring sufficient field resolution and stable convergence of S-parameters. The simulation converged successfully after 7 adaptive passes, with a final delta S below 0.02.

To achieve effective impedance matching and a wide impedance bandwidth, several step-by-step modifications were made to the tapered microstrip line, the ground slot and the spacing between the copper sheets.

### 2.2. Design Process of the Proposed Antenna

**Figure 2:**
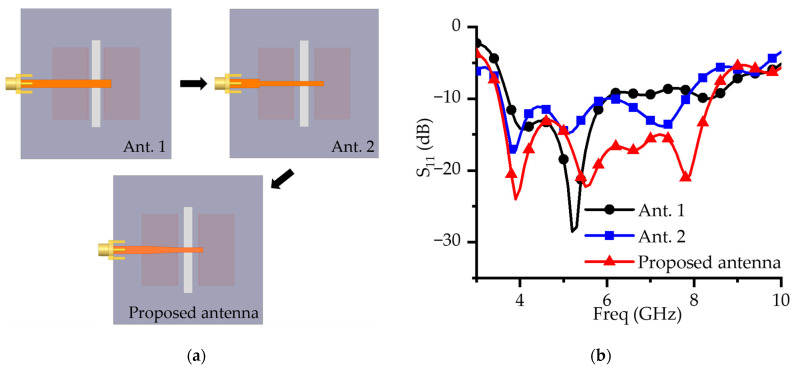
Evolution steps of the ME dipole antenna: (**a**) design evolution in steps; (**b**) S-parameters of the antenna design steps.

Figure 2 illustrates the step-by-step design process for the proposed ME dipole antenna and shows the corresponding S-parameter responses at each stage. By progressively adjusting the gap between the parallel copper plates and refining the geometry of the microstrip feed line, clear improvements in impedance matching and bandwidth are achieved.

Antenna 1 employs a simple, straight microstrip line as the feed. Owing to the abrupt impedance transition between this feed and the radiator, the design exhibits a restricted impedance bandwidth. Antenna 2 introduces a stepped straight-line feed with varying widths at its end. Narrowing the feed line partially mitigates the impedance mismatch between the transmission line and the radiator, raising the effective impedance at the radiator. Consequently, reflection loss is lowered and the impedance bandwidth is moderately extended. Building on this concept, the proposed antenna adopts a linear tapered microstrip line that provides a gradual impedance transition along the feed. This taper greatly diminishes reflections and enhances impedance matching across a wide frequency range.

Figure 2b compares the S-parameter performance of the proposed antenna with that of the two reference designs. Antenna 1 achieves impedance matching only within a limited frequency range of 4–6 GHz, indicating a narrow operational bandwidth. Antenna 2 exhibits improved matching characteristics owing to the stepped feed structure, while the introduction of slot-induced resonances extends the upper passband to 6.1–8 GHz. However, the abrupt width transitions still introduce partial reflections, which compromise the stability of impedance matching across the full operating band. In contrast, the proposed design, which employs a smoothly tapered feed, effectively suppresses such reflections and achieves a wide impedance bandwidth of 84.48% (3.61–8.89 GHz).

These results clearly demonstrate that, compared to the conventional straight and stepped feed structures, the tapered configuration substantially enhances impedance matching, reduces reflection losses and enables broadband performance, thereby validating its effectiveness in ME dipole antenna design.

### 2.3. ME Resonant Mode Analysis

The proposed antenna adopts an ME dipole structure that combines the resonant characteristics of both electric and magnetic dipoles. The fundamental magnetic dipole is generated by a vertically oriented shorted patch of length λ/4, while the electric dipole resonance occurs in a pair of horizontally oriented patches of length λ/2. Additionally, an aperture slot structure is introduced to induce an extra magnetic-dipole resonance [14].

The antenna is designed for a center frequency of approximately 6 GHz (λ ≈ 50 mm), and each component was accordingly optimized based on the operating wavelength. The vertical shorted patch (*H*) is optimized to λ/4 ≈ 12 mm, the horizontal dipole patch (2 × *L*_1_) to λ/2 ≈ 24 mm, and the slot (*L*_2_) to λ/2 ≈ 29 mm.

Within the operating frequency band, the antenna exhibits alternating electric and magnetic-dipole modes. The dominant resonant mode at each frequency is identified through the analysis of equivalent surface current distributions and electric-field distributions. Additionally, the impact of each structural parameter on the resonance frequency is quantitatively evaluated through parametric studies.

**Figure 3:**
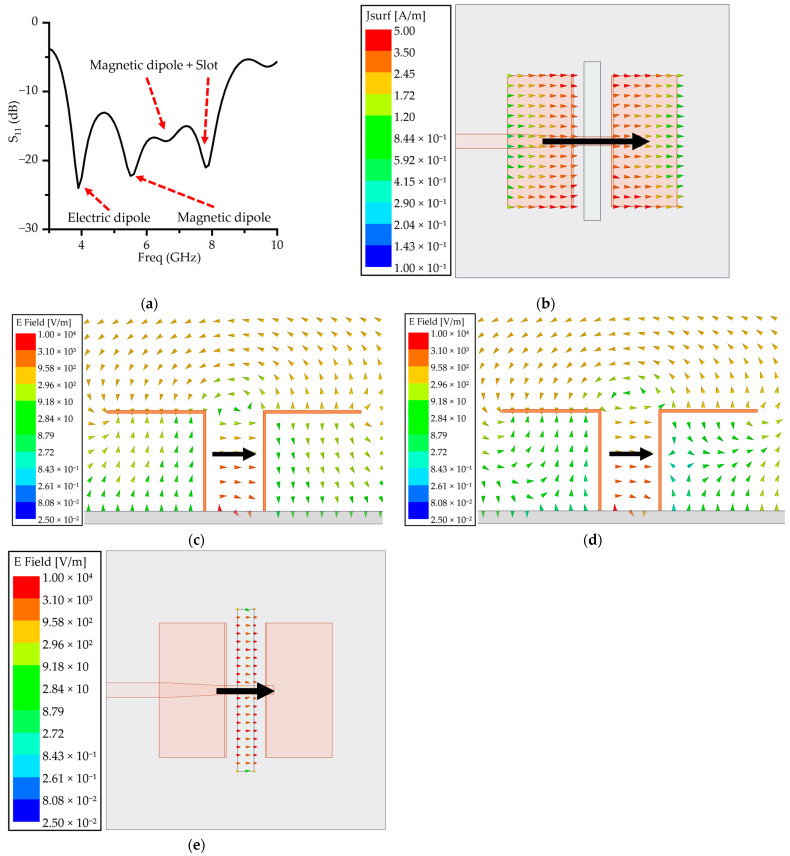
ME resonance field analysis: (**a**) resonant components at each frequency based on simulated S-parameters; (**b**) surface current (J_s_) distribution at 3.9 GHz; (**c**) E-field distribution at 5.5 GHz; (**d**) E-field distribution at 6.3 GHz; (**e**) E-field distribution around the slot at 8 GHz. The black arrows indicate the direction of the electric field distribution.

The electric dipole mode is analyzed through the surface current distribution shown in Figure 3b. In this mode, the current on the horizontal patches predominantly flows in a single direction, concentrating near the center of the dipole. The magnitude and direction of this current exhibit typical dipole behavior, with a periodic pattern over time [21].

**Figure 4:**
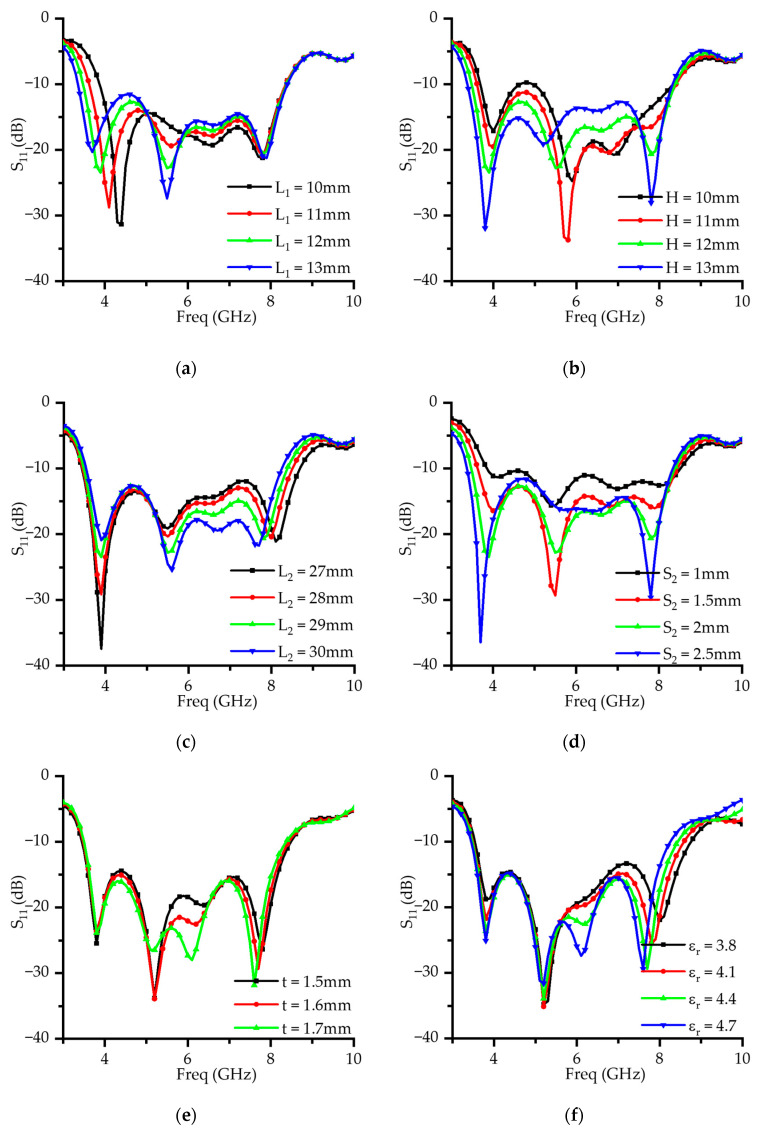
Parametric study of S_11_ at different parameters: (**a**) *L*_1_; (**b**) *H*; (**c**) *L*_2_; (**d**) *S*_2_; (**e**) *t*; (**f**) relative permittivity (ε_r_).

Specifically, the current distribution at 3.9 GHz in Figure 3b reveals a strong concentration near the dipole center, gradually decreasing toward the edges. This is consistent with the current distribution expected from an electric dipole mode. Furthermore, the parametric study for *L*_1_ presented in Figure 4a shows that increasing *L*_1_ from 10 to 13 mm causes a gradual shift in the resonance frequency towards lower values. This trend aligns with standard dipole theory, where an increase in resonant structure length extends the electrical path, thereby reducing the resonance frequency. Taken together, these findings confirm that the electric dipole mode is dominant at 3.9 GHz.

To analyze the magnetic-dipole mode, the electric-field distributions between the shorted patches and around the aperture slot are shown in Figure 3c–e. Magnetic-dipole radiation can be described in terms of the equivalent magnetic surface current (${\vec{J}}_{m}$), defined from the tangential electric field ($\vec{E}$) as

(1) $${\vec{J}}_{m}=-\vec{n}\times \vec{E}$$

when the electric field lies horizontally (along the *x* direction) on a conductor, and its cross-product with the normal vector ($\vec{n}$) produces an equivalent magnetic current flowing in the vertical (*y*) direction. Oppositely directed magnetic currents on facing conductive surfaces form a loop-shaped path, a hallmark of magnetic-dipole resonance [22].

Based on the characteristics of the magnetic-dipole mode, the electric-field distributions at 5.5 and 6.5 GHz (Figure 3c,d) reveal that the electric field between the vertical patches is primarily aligned in the −*x* direction. This distribution creates a strong electric-field difference between the two vertical patches, which induces an equivalent magnetic current forming a loop-shaped path. The current circulates in a closed-loop structure, a behavior that is characteristic of the magnetic-dipole mode and indicates the formation of a magnetic moment through the current loop.

Furthermore, the parametric study of *H*, which is designed to be approximately λ/4, as shown in Figure 4b, demonstrates that increasing *H* leads to a downward shift in both the 5.5 and 6.5 GHz resonant frequencies. This trend aligns with the typical behavior of magnetic-dipole structures, where extending the electrical path increases the effective inductance, thereby reducing the resonant frequency. These results further support the conclusion that the magnetic-dipole mode is dominant in this frequency range.

Additionally, a magnetic-dipole mode is observed around 8 GHz in a similar manner. The electric field generated by the aperture slot, shown in Figure 3e, induces a magnetic loop current circulating around the slot, again exhibiting the typical behavior of magnetic-dipole resonance. The parametric study of *L*_2_ (Figure 4c) reveals that varying the length of the slot causes a significant shift in the resonant frequency near 8 GHz. This confirms that the aperture slot plays a critical role in establishing resonance in this frequency band and indicates that the magnetic-dipole mode is also dominant at this higher frequency.

Moreover, Figure 4d presents the results of the parametric study on *S*_2_, the spacing between the vertical dipoles. The analysis shows that as *S*_2_ decreases, impedance matching deteriorates, leading to a reduction in the overall impedance bandwidth. This implies that *S*_2_ is a key structural parameter influencing the coupling strength between the vertical patches and the radiation efficiency of the magnetic-dipole structure.

As demonstrated, each resonant mode responds sensitively to specific geometric parameters. The underlying nature of each mode, whether electric, magnetic, or slot-induced, can be clearly identified through the surface current and electric-field distributions. These findings verify that the proposed antenna effectively integrates electric, magnetic and slot-based multimode resonant mechanisms, thereby validating its performance as a wideband ME dipole antenna.

### 2.4. ME Resonant Modes: RLC Equivalent Circuit

**Figure 5:**
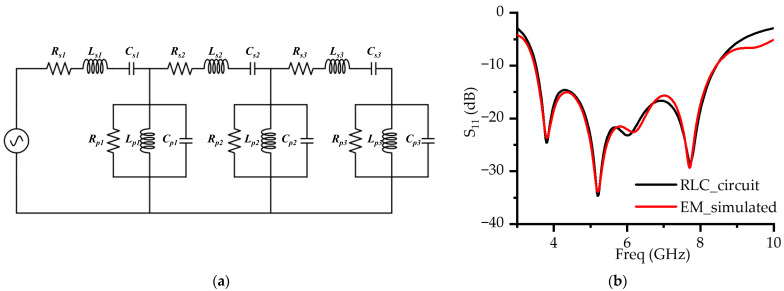
ME resonance RLC equivalent circuit analysis: (**a**) RLC equivalent circuit; (**b**) simulated S_11_ of RLC circuit and EM simulation.

To quantitatively analyze the broadband performance of the proposed ME dipole antenna, an RLC equivalent circuit model was implemented [23]. The peaks and dips in the ME dipole impedance were utilized to model the parallel and series RLC circuits, respectively. The equivalent circuit representation is shown in Figure 5a. Component values were extracted via numerical fitting using Keysight Advanced Design System (ADS) to accurately match the input impedance between the antenna and the equivalent circuit, with the results summarized in Table 2. A comparison of the RLC circuit simulation and the antenna electromagnetic (EM) simulation demonstrates good agreement, as illustrated in Figure 5b.

In particular, the aperture feed structure induces a resonance due to the slot, which produces impedance peaks and forms a parallel resonant subcircuit. This additional resonance significantly contributes to the observed impedance bandwidth enhancement.

## 3. Results

**Figure 6:**
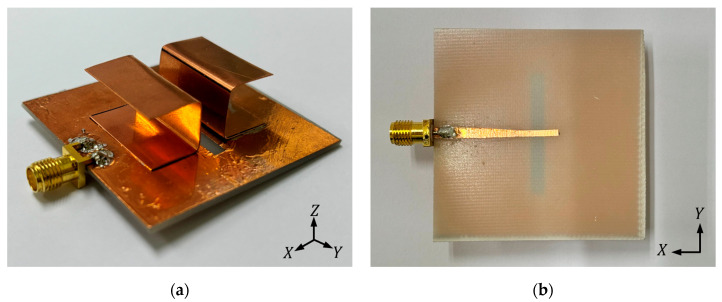
Photographs of the fabricated antenna prototype: (**a**) three-dimensional view of the prototype; (**b**) bottom view of the prototype.

**Figure 7:**
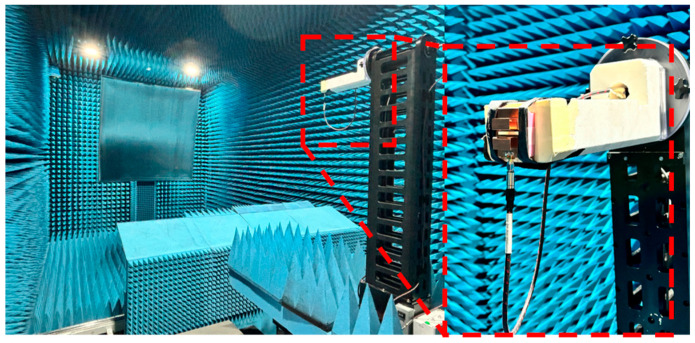
Far-field radiation-pattern measurement setup. The red dashed box is used to enlarge and emphasize the measurement setup.

To verify the performance of the proposed antenna, a prototype was fabricated and tested. Photographs of the prototype are shown in Figure 6, while Figure 7 depicts the millimeter-wave anechoic chamber used for the radiation-pattern measurements. The anechoic chamber used for the measurements has overall dimensions of 8 m (W) × 4 m (D) × 4 m (H). The S-parameters were measured using a vector network analyzer (VNA) over the frequency range of 3–10 GHz. The gain and radiation patterns were evaluated through 2D far-field measurements conducted in the anechoic chamber. The E-plane (φ = 0°) and H-plane (φ = 90°) radiation patterns, corresponding to the ZX and ZY planes, respectively, were measured by sweeping θ from –180° to 180°.

**Figure 8:**
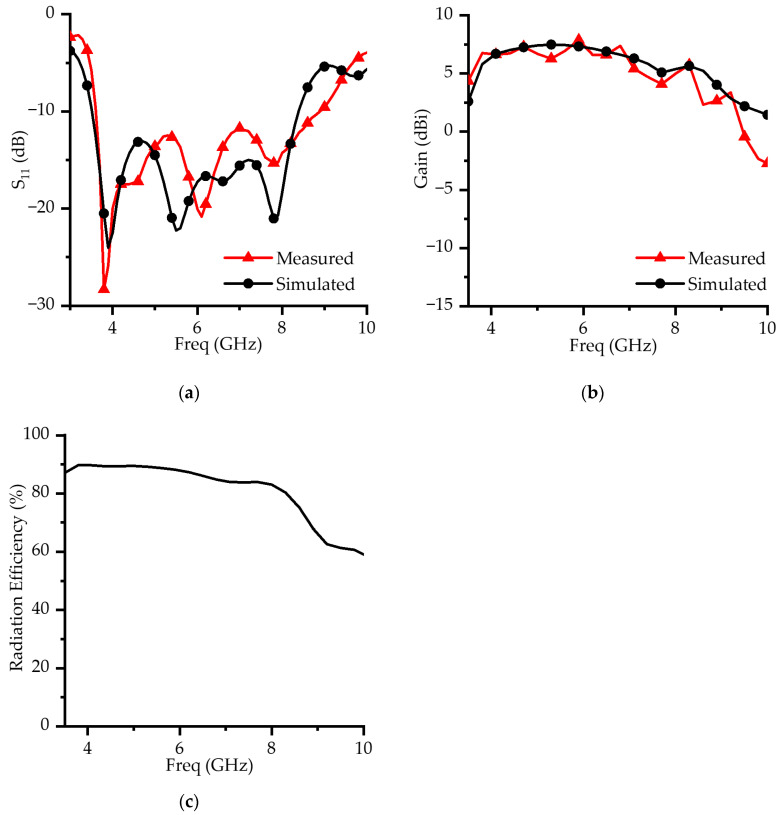
Performance of the proposed antenna: (**a**) simulated and measured S_11_; (**b**) simulated and measured peak gain; (**c**) simulated radiation efficiency.

The measured results across the operating band were directly compared with the simulated data. Figure 8 presents the simulated and measured S_11_ curves, together with the corresponding peak gain and the simulated radiation efficiency over the entire frequency range.

The fabricated prototype achieves an impedance bandwidth of 84.48% (3.61–8.89 GHz) for S_11_ ≤ −10 dB. Good agreement is observed between simulation and measurement at the electric dipole resonance and the aperture slot-induced resonance. In contrast, the magnetic-dipole resonance in the measured data is slightly shifted toward a lower frequency compared to the simulation. Moreover, the measured S_11_ indicates a wider impedance bandwidth than predicted, especially above 8 GHz, where the measured curve shifts toward higher frequencies.

These discrepancies are primarily attributed to the dispersive characteristics of the FR4 substrate and fabrication tolerances [24,25]. The FR4 substrate exhibits frequency-dependent properties, with its relative permittivity decreasing at higher frequencies. The simulations conducted in this study assumed constant dielectric properties and therefore did not fully capture these frequency-dependent variations. Consequently, in the experimental results, resonance frequency shifts and Q-factor degradation occurred, leading to impedance mismatches and increased reflection. A parametric analysis illustrating the effect of variations in the relative permittivity (ε_r_) on the S_11_ characteristics is clearly presented in Figure 4f.

Fabrication tolerances also contribute to the observed differences. As shown in the parametric study (Figure 4), small deviations in H, t and L_2_ result in noticeable changes in resonance and bandwidth. In particular, variations in H and t affect the magnetic-dipole resonance near 6 GHz, while L_2_ influences the bandwidth near 8 GHz. Since these parameters are closely related to the feeding mechanism, they alter the coupling between resonant modes, which explains the frequency shifts in the measured data.

The measured peak in-band gain is 7.88 dBi, showing negligible difference from the simulated value. Additionally, the simulated radiation efficiency remains above 80% throughout most of the operating band. Both gain and radiation efficiency gradually decrease with increasing frequency, primarily due to enhanced dielectric and conductor losses.

As expected for ME dipole antennas, the gain decreases beyond 8 GHz due to the formation of radiation nulls associated with higher-order modes. These results are consistent with the typical behavior of this antenna type and confirm the validity and robustness of the proposed design. Furthermore, the performance degradation at higher frequencies can be attributed to the intrinsic loss characteristics of FR4. Therefore, to improve high-frequency performance, the use of a low-loss substrate such as Rogers is recommended.

**Figure 9:**
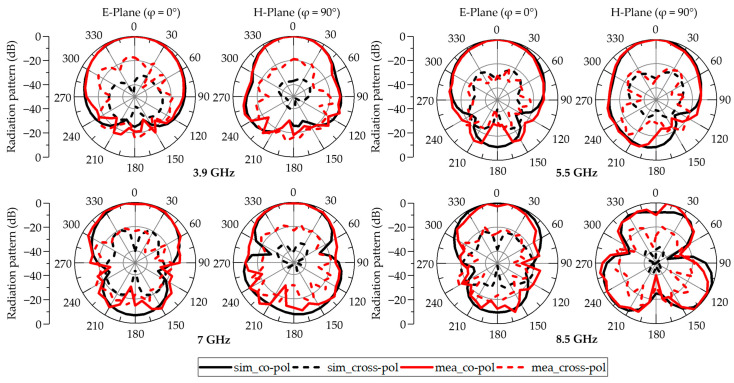
Simulated and measured radiation patterns.

Figure 9 compares the simulated and measured radiation patterns at 3.9, 5.5, 7 and 8.5 GHz. In general, the two sets of patterns correspond well: both the E-plane and H-plane profiles remain symmetrical and preserve stable unidirectional radiation throughout the band. Nonetheless, at the highest test frequency of 8.5 GHz, the measured patterns display some distortion relative to simulation. This deviation is most likely due to the increased sensitivity of the antenna to small fabrication imperfections and to environmental influences that become more pronounced at higher frequencies.

The simulated cross-polarization levels are extremely low. Although the measured cross-polarization is slightly higher, this increase is largely attributable to the weak signal levels involved and to ambient noise in the anechoic chamber. High-frequency coupling effects due to slight misalignments in the measurement setup, particularly in the RF connector and cable, may also contribute to the increase. Additionally, the relatively thick FR4 substrate may excite substrate modes and induce asymmetric current distributions at higher frequencies, leading to pattern distortion and increased cross-polarized radiation. Nevertheless, the measured cross-polarization remains around −15 dB. The discrepancy between simulated and measured cross-polarization patterns thus arises chiefly from the very low amplitude of the cross-polarized signals, which makes them more susceptible to external interference, particularly at the upper end of the band.

## 4. Discussion

Table 3 compares the performance of various aperture excitation-based ME dipole antennas [14,15,17,22,26] reported in the literature with the proposed structure. All compared designs employ slot or aperture-coupling feeding techniques, with additional performance enhancements achieved through various structural approaches such as reflectors, directors, multilayer substrates, or special dielectrics. For instance, ref. [15] incorporated a reflector along with a horn-shaped vertical patch to achieve an exceptionally wide impedance bandwidth of 125%, while ref. [22] employed director loading to realize an 89.3% bandwidth with a high peak gain of 12.3 dBi. Reference [17] enhanced bandwidth through the use of stacked high-permittivity substrates, and ref. [26] improved antenna performance by introducing a liquid-dielectric resonator. Although [14] attained a wide bandwidth of 85% and a peak gain of 8 dBi, its relatively large volume of (1.74 × 1.74 × 0.26) $\lambda_{0}^{3}$ poses challenges for integration into compact systems.

In contrast, the antenna proposed in this work adopts a much simpler configuration, comprising only a single-layer planar patch and a vertical metal patch. Despite its structural simplicity, it achieves a broad impedance bandwidth of approximately 84.48% and a peak gain of 7.88 dBi, while successfully reducing the antenna volume to (1.04 × 1.04 × 0.28) $\lambda_{0}^{3}$. This performance is realized without the use of additional directors, reflectors, multilayer substrates, or special dielectric materials. When compared to designs such as the high-permittivity stacked configuration in [17] or the liquid-dielectric-based antenna in [26], the proposed design offers a significantly simplified fabrication process. It is compatible with standard PCB manufacturing techniques, providing a high degree of manufacturability and practical feasibility for real-world deployment.

A comparative analysis was conducted between the proposed ME dipole and previously reported Vivaldi antennas [27,28] in terms of bandwidth, gain and size. Vivaldi-based designs offer significantly wider impedance bandwidth and higher gain, but they generally require larger vertical dimensions and involve more complex structures. In contrast, the proposed ME dipole features a smaller form factor and a simpler design while maintaining a comparable gain. This comparison highlights the trade-off between bandwidth and structural simplicity among UWB antennas (ME dipole and Vivaldi).

Overall, the proposed design effectively balances three often-competing design goals: wide bandwidth, structural simplicity and compactness. While the peak gain is marginally lower than that of some high-gain designs, this is a reasonable and practical trade-off in favor of improved integration potential and ease of fabrication. Compared to existing antennas using similar aperture excitation methods, the proposed structure demonstrates an excellent balance between performance, physical size and implementation complexity. Additionally, the antenna’s operation across the 3.1–10.6 GHz ultra-wideband range highlights its potential for next-generation 6G applications, including high-precision localization, advanced sensing and low-power short-range wireless communications.

## 5. Conclusions

In this study, a broadband ME dipole antenna was proposed, employing a tapered microstrip line and a ground-etched aperture excitation. The slot structure etched into the ground plane introduces an additional magnetic resonance, while the tapered microstrip line facilitates efficient impedance matching across multiple resonant modes, resulting in a wide operating bandwidth. To validate the design, a prototype antenna was fabricated and experimentally evaluated. The measured results demonstrated an impedance bandwidth of 84.48% (3.61–8.89 GHz) for $S_{11}$ ≤ −10 dB, along with a maximum in-band gain of 7.88 dBi. Furthermore, the antenna maintained stable unidirectional radiation patterns throughout the entire frequency range, confirming its suitability for broadband applications, such as those required in 5G wireless communication systems. The proposed ME dipole unit, with its wideband capabilities and structurally simple design, presents a promising solution for future broadband wireless systems. This includes emerging 6G technologies such as reflect arrays and intelligent reflecting surfaces (IRS), where compact, high-performance antennas are essential.

## Figures and Tables

**Table 1:** Parameters of proposed antenna.

Parameter	*G*	*t*	*L*_1_	*L*_2_	*L*_3_	*L*_4_	*L*_5_
Value (mm)	50	1.6	12	29	11	10	9
$\lambda_{0}$ *	1	0.032	0.24	0.58	0.22	0.2	0.18
Parameter	*H*	*W*_1_	*W*_2_	*W*_3_	*S*_1_	*S*_2_	
Value (mm)	12	24	2.6	1.6	3	2	
$\lambda_{0}$ *	0.24	0.48	0.052	0.032	0.06	0.04	

* *λ*_0_ is the free-space wavelength at the center frequency of 6 GHz.

**Table 2:** Component values of the equivalent circuit.

Element	Value	Element	Value	Element	Value
R_s1_	11.1 Ω	R_s2_	24.8 Ω	R_s3_	1 Ω
L_s1_	1.43 nH	L_s2_	1.83 nH	L_s3_	3.9 nH
C_s1_	0.58 pF	C_s2_	0.452 pF	C_s3_	0.935 pF
R_p1_	2500 Ω	R_p2_	24.8 Ω	R_p3_	141.5 Ω
L_p1_	0.721 nH	L_p2_	0.158 nH	L_p3_	3.45 nH
C_p1_	1.21 pF	C_o2_	5.73 pF	C_p3_	0.75 pF

**Table 3:** Performance comparison of aperture-excited ME dipole and other UWB antennas.

Ref.	Imp.BW (GHz/%)	Peak Gain (dBi)	Size $\left(mm^{3},\lambda_{0}^{3}\right)$	Bandwidth Enhancement Technique
[14]	2.5–6.2 (85)	8	120 × 120 × 18, $(1.74\;\times \;1.74\;\times \;0.26)\lambda_{0}^{3}$	/
[15]	2.24–10 (125)	7.9	68 × 68 × 18, $(1.39\;\times \;1.39\;\times \;0.37)\lambda_{0}^{3}$	Reflector + Horn-shaped vertical structure
[17]	2.35–4.27 (58)	7.8	70 × 70 × 24.28, $(0.77\;\times \;0.77\;\times \;0.27)\lambda_{0}^{3}$	High-permittivity stacked substrate
[22]	2.35–6.14 (89.3)	12.3	155 × 155 × 33.3, $(2.19\;\times \;2.19\;\times \;0.47)\lambda_{0}^{3}$	Novel director loaded
[26]	2.45–5.3 (73.5)	7	52 × 52 × 13.6, $(0.66\;\times \;0.66\;\times \;0.16)\lambda_{0}^{3}$	Liquid-dielectric resonator
[27]	3.7–18 (131.8)	6.9	42 × 36 × 1.6, $(1.52\;\times \;1.3\;\times \;0.06)\lambda_{0}^{3}$	Balanced antipodal Vivaldi antenna (BAVA)
[28]	3.1–10.6 (109)	10	35 × 35 × 103, $(0.8\;\times \;0.8\;\times \;2.35)\lambda_{0}^{3}$	Vivaldi antenna + Novel director loaded
This work	3.61–8.89 (84.48)	7.88	50 × 50 × 13.6, $(1.04\;\times \;1.04\;\times \;0.28)\lambda_{0}^{3}$	/

*λ*_0_ denotes the free space wavelength at the center operating frequency.

## Data Availability

The data presented in this study are available on request from the corresponding author.
